# Supplementary material for: Chimeric MerR-Family Regulators and Logic Elements for the Design of Metal Sensitive Genetic Circuits in Bacillus subtilis
Source: ACS Synth Biol. 2023 Jan 11;12(3):735–49. doi: 10.1021/acssynbio.2c00545 (PMC10028694; doi:10.1021/acssynbio.2c00545)
Supplement: Supplementary file 1 — sb2c00545_si_001.pdf [file sb2c00545_si_001.pdf]

Supplemental Figures and Tables for:

Chimeric MerR-Family Regulators and Logic Elements for the Design of Metal Sensitive Genetic Circuits in Bacillus subtilis

Jasdeep S. Ghataora, Susanne Gebhard*, Bianca J. Reeksting*

Life Sciences Department, Milner Centre for Evolution, University of Bath, Claverton Down, Bath BA2 7AY, United Kingdom

*Corresponding authors:

Bianca Reeksting (bjr39@bath.ac.uk)

Susanne Gebhard (sg844@bath.ac.uk)

Supplementary Table S1. Bacterial strains used within this study 2

Supplementary Table S2. Plasmids used in this study 4

Supplementary Table S3. Primers and oligonucleotides used in this study 7

Supplementary Table S4. Relevant DNA sequences for plasmid construction 11

Supplementary Figure S1. Comparison of Gram +ve and Gram -ve MerR promoter activity in *B. subtilis* 15

Supplementary Figure S2. Comparison of ZntR sequences and structural analysis of the chimera MerRZntR 16

Supplementary Figure S3. Activity of double and triple MerRZntR mutants against the wild-type and single mutant MerRZntR^{A29E} 17

Supplementary Figure S4. Comparison of inter-domain communication between CueR and the chimera MerRCueR 18

Supplementary Figure S5. Dose response of P$_{merR}$ regulated by MerRCueR^{mut3} in response to Ag^+ induction 19

Supplementary Figure S6. Wild-type (P$_{merR20}$) and mutant (P$_{merR19}$) promoters 20

Supplementary Figure S7. The *B. subtilis* SANDBOX plasmids 21

Supplementary Figure S8. *Bacillus subtilis* natively regulated CzrA based circuit 22

References 23

Supplementary Table S1. Bacterial strains used in this study

**Strain**	**Characteristics**[a]	**Construction**[b]
DH5α	*Escherichia coli* DH5α	Laboratory stock
W168	Wild-type *Bacillus subtilis* W168	Laboratory stock
PAO1	Wild-type *Pseudomonas aeruginosa* PAO1	Laboratory stock
SGB1003	W168 *sacA*:: pJGlux01-P_{merR20}-*luxABCDE*-cm^{r}	pJGlux01 -> W168
SGB1005	W168 *sacA*:: pJGlux01-P_{merR20}-*luxABCDE*-cm^{r}; *thrC*::pJGXT01- P_{xylA}-MerR- spc^{r}	pJGXT01 -> SGB1003
SGB1006	W168 sacA::pJGlux03-P_{veg}-*luxABCDE*-cm^{r}	pJGlux03 -> W168
SGB1007	W168 *sacA*::pAH328	pAH328 -> W168
SGB1011	W168 *sacA*:: pJGlux01-P_{merR20}-*luxABCDE*-cm^{r}; *thrC*::pJGXT02- P_{xylA}-MerRZntR- spc^{r}	pJGXT02 -> SGB1003
SGB1014	W168 *sacA*::pJGlux02- P_{merR19}-*luxABCDE*-cm^{r}	pJGlux02 -> W168
SGB1015	W168 *sacA*:: pJGlux02- P_{merR19}-*luxABCDE*-cm^{r}; *thrC*:: pJGXT01- P_{xylA}-MerR- spc^{r}	pJGXT02 ->SGB1014
SGB1027	W168 *sacA*:: pJGlux01-P_{merR20}-*luxABCDE*-cm^{r} -cm^{r}; *thrC*:: pJGXT03- P_{xylA}-MerRCueR -spc^{r}	pJGXT03 -> SGB1003
SGB1028	W168 *sacA*:: pJGlux01-P_{merR}-*luxABCDE*-cm^{r}; *thrC*:: pJGXT07- P_{xylA}-MerRCueR^{A29T} -spc^{r}	pJGXT07 -> SGB1003
SGB1029	W168 *sacA*:: pJGlux01-P_{merR20}-*luxABCDE*-cm^{r}; *thrC*:: pJGXT08- P_{xylA}-MerRCueR$^{A29T/G30P}$ -spc^{r}	pJGXT08 -> SGB1003
SGB1030	W168 *sacA*:: pJGlux01-P_{merR20}-*luxABCDE*-cm^{r}; *thrC*:: pJGXT11-P_{xylA}-MerRCueR$^{A29T/G30P/P32M}$ -spc^{r}	pJGXT11 -> SGB1003
SGB1034	W168 sacA::pJGlux04-P_{cadA}-*luxABCDE*-cm^{r}	pJGlux04 -> W168
SGB1035	W168 *sacA*:: pJGlux01-P_{merR20}-*luxABCDE*-cm^{r}; *thrC*:: pJGXT15-P_{xylA}-MerRZntR^{A29E} -spc^{r}	pJGXT15 -> SGB1003
SGB1036	W168 *sacA*:: pJGlux01-P_{merR20}-*luxABCDE*-cm^{r}; *thrC*:: pJGXT16- P_{xylA}-MerRZntR$^{A29E/G30H}$ -spc^{r}	pJGXT16 -> SGB1003
SGB1047	W168 *sacA*:: pJGlux01-P_{merR20}-*luxABCDE*-cm^{r}; *thrC*:: pJGXT23-P_{xylA}-MerRZntR$^{A29E/G30H/P32V}$ -spc^{r}	pJGXT23 -> SGB1003
SGB1050	W168 *sacA*:: pJGlux02-P_{merR19}-*luxABCDE*-cm^{r}; *thrC*:: pJGXT11-P_{xylA}-MerRCueR$^{A29T/G30P/P32M}$ -spc^{r}	pJGXT11 -> SGB1014
SGB1060	W168 *ΔoxdC-yvrJ-rsiO/sigO-rsoA*	pBSANDdel -> W168
SGB1061	W168 *ΔoxdC-yvrJ-rsiO/sigO-rsoA*; *sacA*::pBSANDlux-cm^{r}	pBSANDlux -> SGB1060
SGB1064	W168 *ΔoxdC-yvrJ-rsiO/sigO-rsoA*; *sacA*::pBSANDlux-cm^{r}; *lacA*::pBSAND2-P_{xylA}-MLS^{r}	pBSAND2-P_{xylA} -> SGB1061
SGB1065	W168 *ΔoxdC-yvrJ-rsiO/sigO-rsoA*; *sacA*::pBSANDlux-cm^{r}; *lacA*::pBSAND2-P_{xylA}-MLS^{r}; *thrC*::pBSAND1-P_{liaI}-spc^{r}	pBSAND1-P_{liaI} -> SGB1064
SGB1067	W168 *sacA*::pBSGGlux-P_{cadA}-cm^{r}	pBSGGlux-P_{cadA} -> W168
SGB1068	W168 *ΔoxdC-yvrJ-rsiO/sigO-rsoA*; *sacA*::pBSANDlux -cm^{r}; *lacA*::pBSAND2-P_{cadA}-MLS^{r}	pBSAND2-P_{cadA} -> SGB1061
SGB1072	W168 *ΔoxdC-yvrJ-rsiO/sigO-rsoA*; *sacA*::pBSANDlux -cm^{r}; *lacA*::pBSAND2-P_{cadA}-	pBSAND1-P_{xylA}-MerRZntR^{A29E}-Terminator-P_{merR}-> SGB1068

	MLS^r; *thrC*::pBSAND1-P$_{xylA}$-MerRZntR^{A29E}-Terminator-P$_{merR20}$-*spc*^{r}	

[a] Relevant characteristics are listed. Antibiotic resistance cassettes are denoted as follows: *cm*: chloramphenicol resistance; spc: spectinomycin resistance; MLS: erythromycin and lincomycin resistance.

[b] The direction of strain construction is indicated by an arrow which involves transformation with plasmids as indicated. Plasmids referred to are given in Table S2.

Supplementary Table S2. Plasmids used in this study

Plasmid	Characteristics[a]	Source
pXT	Plasmid for xylose-inducible gene expression; integrates in *thrC*; spc^r, amp^r, ori ColE1	[1]
pAH328	Plasmid for transcriptional promoter fusions to *luxABCDE* (luciferase); integrates at *sacA*; cm^r, amp^r	[2]
pBS4S	Empty plasmid, integration at *thrC*, amp^r, spec^r,, ori ColE1	[3]
pBS2E	Empty plasmid, integration at *lacA*, amp^r, *mls*^{r}, ori ColE1	[3]
pBS3Clux	Bacterial luciferase (*luxABCDE*) plasmid, integration at *sacA*, amp^r, cm^r, ori ColE1	[3]
pSB1A3-P_{xylA}	*amp*^{r} , ori pMB1, P_{xylA} (xylose inducible promoter)	[3]
pSB1C3-P_{veg}	*cm*^{r} *, ori pMB1,* P_{veg} (strong constitutive B. subtilis promoter)	[3]
pJOE8999	CRISPR-Cas9 deletion plasmid, *kan*^{r}; pUC-ori , temperature sensitive ori for *B. subtilis*(permissive at 30°C)	[4]
pJGlux01	P_{merR20} – *luxABCDE* transcriptional fusion, derived from pAH328	This study
pJGlux02	P_{merR19} – *luxABCDE* transcriptional fusion, derived from pAH328	This study
pJGlux03	P_{veg}-*luxABCDE* transcription fusion, derived from pAH328	This study
pJGlux04	P_{cadA19}-*luxABCDE* transcriptional fusion, derived from pAH328	This study
pJGXT01	P_{xylA} -MerR transcriptional fusion, derived from pXT	This study
pJGUC01	pUC19 derived vector, *amp*^{r}, ori ColE1, source of MerRZntR chimera RBS and CDS	This study (GenScript synthesised vector)
pJGXT02	P_{xylA} -MerRZntR transcriptional fusion, derived from pXT	This study
pJGUC02	pUC19 derived vector, *amp*^{r}, ori ColE1, source of MerRCueR chimera RBS and CDS	This study (GenScript synthesised vector)
pJGXT03	P_{xylA} -MerRCueR transcriptional fusion, derived from pXT	This study

pJGXT07	P_{xylA} -MerRCueR^{A29T} transcriptional fusion, derived from pJGXT03	This study
pJGXT08	P_{xylA} -MerRCueR$^{A29T/G30P}$ transcriptional fusion, derived from pJGXT03	This study
pJGXT11	P_{xylA} -MerRCueR$^{A29T/G30P/P32M}$ transcriptional fusion, derived from pJGXT03	This study
pJGXT15	P_{xylA} -MerRZntR^{A29E} transcriptional fusion, derived from pJGXT02	This study
pJGXT16	P_{xylA} -MerRZntR$^{A29E/G30H}$ transcriptional fusion, derived from pJGXT02	This study
pJGXT23	P_{xylA} -MerRZntR$^{A29E/G30H/P32V}$ transcriptional fusion, derived from pJGXT02	This study
pJGBS3Clux02	Bacterial luciferase (*luxABCDE*) plasmid with BsaI deletion in *amp*^{r}, pBS3Clux derived	This study
pJGBS3Clux03	Bacterial luciferase (*luxABCDE*) plasmid with BsaI deletion in *amp*^{r} and *luxC*, derived from pJGBS3Clux02	This study
pBSGGlux	Bacterial luciferase (*luxABCDE*) plasmid, integration at *sacA*, amp^r, cm^r, ori ColE1 – Golden Gate vector, derived from pJGBS3Clux03	This study
pJGBS2E03	Empty plasmid with BsaI deletion in *amp*^{r} , derived from pBS2E	This study
pJGBS2E04	Empty plasmid with BsaI deletion in *amp*^{r} , and NgoMIV insertion in *amp*^{r} derived from pBS2E	This study
pBSAND2	Empty plasmid with Golden Gate cloning site allowing for SigO expression, derived from pJGBS2E04	This study
pJGBS4S01	Empty plasmid with BsaI deletion in *amp*^{r}	This study
pBSAND1	Empty plasmid with Golden Gate cloning site allowing for RsoA expression, derived from pJGBS4S01	This study
pBSANDdel	CRISPR-Cas9 deletion vector allowing for guided cut upstream of *rsiO* in *B. subtilis* with repair homology flanking	This study

	region to allow for *oxdC-yvrJ-rsiO/sigO-rsoA* deletion (SigO-RsoA regulon)	
pBSANDlux	P_{oxdC} – luciferase reporter regulated by both SigO and RsoA, derived from pBSGGlux	This study
pBSAND2-P_{xylA}	Xylose-inducible expression of SigO, derived from pBSAND2	This study
pBSAND1-P_{liaI}	Bacitracin-inducible expression of RsoA, derived from pBSAND1	This study
pBSGGlux-P_{cadA}	Metal-inducible expression of luciferase – natively regulated by CzrA in *B. subtilis*	This study
pBSAND2-P_{cadA}	Metal-inducible expression of SigO, derived from pBSAND2	This study
pBSAND1-P_{xylA}-MerRZntR^{A29E}-Terminator-P_{merR20}	Xylose-inducible expression of MerRZntR^{A29E} to subsequently regulate RsoA expression driven by P_{merR20} (regulated by MerRZntR^{A29E}) – derived from pBSAND1	This study

[a] Relevant characteristics are listed. Antibiotic resistance cassettes as follows: *amp*^{r} ; ampicillin resistance; *kan*^{r} : kanamycin resistance; *cm*^{r} : chloramphenicol resistance; spc^r : spectinomycin resistance; *mls*: erythromycin and lincomycin resistance.

Supplementary Table S3. Primers and oligonucleotides used in this study

Primer	Description	Sequence[a]	Function
SG0245	pBS2E/pBSAND2 check fwd	GGCAACCGAGCGTTCTG	Verification of inserts into pBS2E derived plasmids
SG0246	pBS2E/pBSAND2 check rev	CTGACAGCGTTTCGATCC	Verification of inserts into pBS2E derived plasmids
SG0528	*sacA* insertion up fwd	CTGATTGGCATGGCGATTGC	Verification of *sacA* integration for pBS3Clux derived plasmids
SG0529	*sacA* insertion up rev	ACAGCTCCAGATCCTCTACG	Verification of *sacA* integration for pBS3Clux derived plasmids
SG0530	*sacA* insertion down fwd	GTCGCTACCATTACCAGTTG	Verification of *sacA* integration for pBS3Clux derived plasmids
SG0531	*sacA* insertion down fwd	TCCAAACATTCCGGTGTTATC	Verification of *sacA* integration for pBS3Clux derived plasmids
SG0148	pBS2E/pBSAND2 up fwd	GCATACCGGTTGCCGTCATC	Verification of lacA integration for pBS2E derived plasmids
SG1305	pBS2E/pBSAND2 up rev	ATCTATTATTTAACGGGAGGAAATAATTC	Verification of lacA integration for pBS2E derived plasmids
SG1346	pBS2E/pBSAND2 down fwd	CTGCAGAGATATCGATTTCAAGC	Verification of lacA integration for pBS2E derived plasmids
SG0149	pBS2E/pBSAND2 down fwd	GAACTACATGCACTCCACAC	Verification of lacA integration for pBS2E derived plasmids
SG601	pBS4S/pBSAND1 check fwd	CAGTCAACCCTTACCGCATTG	Verification of inserts into pBS4S derived plasmids
SG602	pBS4S/pBSAND1 check fwd	CCTCCTCACTATTTTGATTAGTACC	Verification of inserts into pBS4S derived plasmids
SG0985	EcoRI + P_{merR} fwd	tttaa**GAATTC**AACGGAAGAATGTGGCTTCTTGG	Amplification of P_{merR20}
SG0986	SpeI + P_{merR} rev	tttaa**ACTAGT**TGATTTTCATCCCCATATTGTCACC	Amplification of P_{merR20}
SG0987	BamHI + Native RBS + MerR fwd	tttaa**GGATCC**<u>gaggt</u>GACAATATGGGGATGAAAATC	Amplification of MerR with native RBS
SG0988	EcoRI + MerR rev	tttaa**GAATTC**TTATTTATCAGGCCCTCCCATTAACGTT	Amplification of MerR with native RBS
SG0991	pAH328/pBS3Clux check fwd	GAGCGTAGCGAAAAATCC	Verification of inserts into pAH328/pBS3Clux derived plasmids
SG0992	pAH328/pBS3Clux check rev	GAAATGATGCTCCAGTAACC	Verification of inserts into pAH328/pBS3Clux derived plasmids
SG1124	P_{merR20} 1-bp deletion fwd	GGTACAGGGTTATACTTTTTATTGAGGTGACAATATGGGG	Mutagenesis of P_{merR20} make P_{merR19}
SG1125	P_{merR20} 1-bp deletion fwd	AAAAAGTATAACCCTGTACCATAGTACACGGTCAAGTCAT	Mutagenesis of P_{merR20} make P_{merR19}

SG1142	MerRCueR^{A29T} fwd	AGGATTGATAACAGGGCCTCCCAGAAACG AATCAGGGTATCGC	MerRCueR -> MerCueR^{A29T} mutagenesis
SG1143	MerRCueR^{A29T} rev	TGGGAGGCCCTGTTATCAATCCTTTCCGCT CGTAATACCGAAC	MerRCueR -> MerCueR^{A29T} mutagenesis
SG1154	MerRCueR$^{A29T/G30P}$ fwd	ATTGATAACACCGCCTCCCAGAAACGAAT CAGGGTATCGCACC	MerRCueR -> MerRCueR$^{A29T/G30P}$ mutagenesis
SG1155	MerRCueR$^{A29T/G30P}$ rev	TTCTGGGAGGCGGTGTTATCAATCCTTTCC GCTCGTAATACCG	MerRCueR -> MerRCueR$^{A29T/G30P}$ mutagenesis
SG1164	P_{cadA19} fwd	TTTAA**GAATTC**TTCGCGCTCGTAGTAGCG GATGGTC	Amplification of the P_{cadA19} promoter from *P. aeruginosa*
SG1171	P_{cadA19} fwd	TTTAA**ACTAGT**TGTTGGGTGCGATTGCAC ACCCTGT	Amplification of the P_{cadA19} promoter from *P. aeruginosa*
SG1167	MerRCueR$^{A29T/G30P/P32M}$ fwd	AACACCGCCTATGAGAAACGAATCAGGGT ATCGCACCTACACG	MerRCueR$^{A29T/G30P}$ -> MerRCueR$^{A29T/G30P/P32M}$ mutagenesis
SG1168	MerRCueR$^{A29T/G30P/P32M}$ rev	ATTCGTTTCTCATAGGCGGTGTTATCAATC CTTTCCGCTCGTA	MerRCueR$^{A29T/G30P}$ -> MerRCueR$^{A29T/G30P/P32M}$ mutagenesis
SG1172	MerRZntR^{A29E} fwd	AGGATTGATAGAAGGGCCTCCCAGAAAC GAATCAGGGTATCGA	MerRZntR -> MerRZntR^{A29E} mutagenesis
SG1173	MerRZntR^{A29E} rev	TGGGAGGCCCTTCTATCAATCCTTTCCGCT CGTAATACCGAAC	MerRZntR -> MerRZntR^{A29E} mutagenesis
SG1174	MerRZntR$^{A29E/G30H}$ fwd	GAAAGGATTGATAGAACATCCTCCCAGAA ACGAATCAGGGTATCGACTA	MerRZntR -> MerRZntR$^{A29E/G30H}$ mutagenesis
SG1175	MerRZntR$^{A29E/G30H}$ rev	CGTTTCTGGGAGG**ATGTTC**TATCAATCCTT TCCGCTCGTAATACCGAAC	MerRZntR -> MerRZntR$^{A29E/G30H}$ mutagenesis
SG1200	MerRZntR$^{A29E/G30H/P32V}$ fwd	CGAGCGGAAAGGATTGATAGAACATCCTG TTAGAAACGAATCAGGGTATCGACTATAT ACC	MerRZntR -> MerRZntR$^{A29E/G30H/P32V}$ mutagenesis
SG1201	MerRZntR$^{A29E/G30H/P32V}$ rev	CGATACCCTGATTCGTTTCTAACAGGATGT TCTATCAATCCTTTCCGCTCGTAATACC	MerRZntR -> MerRZntR$^{A29E/G30H/P32V}$ mutagenesis
SG1239	BsaI deletion in *luxC* fwd	GACGGAATGAGGCCGTTGCAACGATTAGT GACATATATT	BsaI deletion in *luxC* of pBS3Clux
SG1240	BsaI deletion in *luxC* rev	GTTGCAACGGCCTCATTCCGTCATGAGATC CACCAACTC	BsaI deletion in *luxC* of pBS3Clux
SG1242	BsaI deletion in *amp^r* fwd	TGAGCGTGGCTCTCGCGGTATCATTGCAG CACTGG	BsaI deletion in *amp^r*
SG1243	BsaI deletion in *amp^r* rev	ACCGCGAGAGCCACGCTCACCGGCTCCAG ATTTATCAG	BsaI deletion in *amp^r*
SG1245	NgoMIV insertion in *amp^r* fwd	TTGACGCCGGCCAAGAGCAACTCGGTCGC CGCATAC	Insertion of NgoMIV site in *amp^r*
SG1246	NgoMIV insertion in *amp^r* rev	GTTGCTCTTGGCCGGCGTCAACACGGGAT AATACC	Insertion of NgoMIV site in *amp^r*
SG1274	SfiI + RBS + SigO fwd	TTAAA**GGCC**ACCCA**GGCC**gctcttaaggagga ttttagaATGAAGCATCCCATCGTGAAGCAT	Amplification of SigO to include RBS R1

SG1248	PstI + SigO + 3' - Terminator rev	AAA**CTGCAG***AAAAAACGCGAGCGTCAGGCGCTCGCGTTTTT*CTAATCGATTACTTTTCCCCCTTTTTTTGCGC	Amplification of SigO to include 3'-Terminator
SG1275	SfiI + RBS + RsoA fwd	TTAAA**GGCC**ACCCA**GGCC**gctcttaaggaggattttagaGTGGACGGCCAGTTTGAACAAAA	Amplification of RsoA to include RBS R1
SG1250	PstI + RsoA + 3'-Terminator rev	TTAAA**CTGCAG**AAAAAACGCGAGCGTCAGGCGCTCGCGTTTTTCTAATCGATTATGAGTTCTCATCTACCATGTAC	Amplification of RsoA to include 3' - Terminator
SG1272	EcoRI + RFP + 5'-Terminator + 5'-BsaI fwd	TTAAA**GAATTC**AAAAACGCGAGCGCCTGACGCTCGCGTTTTTTCC**C**TACTC**GAGACC**TCATTCTCGCAATACGCAAACCGCCTCTC	Amplification of RFP to include 5'-Terminator and 5' BsaI site (new BsaI Golden Gate site) – For plasmids pBSAND1/2 and pBSANDlux
SG1273	SfiI + RFP + 5'-BsaI site rev	TTAAA**GGCC**TGGGT**GGCC**TACTGATAATTCT**GAGACC**ATAAACGCAGAAAGGCCCACCC	Amplification of RFP to include 3'-BsaI site (new BsaI Golden Gate site) – For plasmids pBSAND1/2
SG1324	PstI + RFP + 3'- BsaI site rev	TTAAA**CTGCAG**GGTACTGATAATTCT**GAGACC**ATAAACGCAGAAAGGCCCACCC	Amplification of RFP to include 3'-BsaI site (new Golden Gate site) – for plasmid pBSANDlux
SG1297	BsaI + P_{xylA} fwd	TTTAAA**GGTCTC**ATACTAAGGCCAAAAAACTGCTGCCTTCG	Amplification of P_{xylA}
SG1298	BsaI + P_{xylA} rev	TTTAAA**GGTCTC**TATTCTTCGATAAGCTTGGGATCCCAG	Amplification of P_{xylA}
SG1299	BsaI + P_{oxdC} fwd	TTTAAA**GGTCTC**ATACTGATTCGAAAAGAAGTTTGATCA	Amplification of P_{oxdC}
SG1300	BsaI + P_{oxdC} rev	TTTAAA**GGTCTC**TATTCTTCGTTTTCCCTTCTGTTTTGT	Amplification of P_{oxdC}
SG1303	pBSGGlux new GoldenGate site check fwd	AAAAACGCGAGCGCCTGAC	Check for insertion of RFP with new Golden Gate site in pBSGGlux
SG1325	pBSGGlux new GoldenGate site check rev	GCAGGGTACTGATAATTCTGAGACCAT	Check for insertion of RFP with new Golden Gate site in pBSGGlux
SG1326	*oxdC-yvrJ-rsiO/sigO-rsoA* left hand homology + SfiI fwd	Ttaaa**GGCC**AACGA**GGCC**TCAATGAAGCTGTCAGAGATGAAC	Amplification of *oxdC-yvrJ-rsiO/sigO-rsoA*left hand homology region for pBSANDdel deletion vector
SG1327	*oxdC-yvrJ-rsiO/sigO-rsoA* left hand homology + SfiI rev	TTAAA**GGCC**TAAAT**GGCC**AAGAACGCATTCCAATTGGATGC	Amplification of *oxdC-yvrJ-rsiO/sigO-rsoA* left hand homology region for pBSANDdel deletion vector
SG1328	*oxdC-yvrJ-rsiO/sigO-rsoA* right hand homology + SfiI fwd	TTAAA**GGCC**ATTTA**GGCC**TTTCATATGAAAAAACATCAAAAGGGAG	Amplification of *oxdC-yvrJ-rsiO/sigO-rsoA* right hand homology region for pBSANDdel deletion vector

SG1329	*oxdC-yvrJ-rsiO/sigO-rsoA* right hand homology + SfiI rev	TTAAA**GGCC**TTATT**GGCC**GCCTACGATCA ACTCGGCATT	Amplification of *oxdC-yvrJ-rsiO/sigO-rsoA* right hand homology region for pBSANDdel deletion vector
SG1344	*ΔoxdC-yvrJ-rsiO/sigO-rsoA* deletion check fwd	GAACATATTCGAATCTCCTTTCAA	Check for *ΔoxdC-yvrJ-rsiO/sigO-rsoA* deletion using pBSANDdel
SG1345	*ΔoxdC-yvrJ-rsiO/sigO-rsoA* deletion check rev	TGCCTTCAGGATTTGGGATAAA	Check for *ΔoxdC-yvrJ-rsiO/sigO-rsoA* deletion using pBSANDdel
SG1347	pBSANDdel homology insertion check fwd	GATTTGTTCAGAACGCTCGGT	Check for insertion of *oxdC-yvrJ-rsiO/sigO-rsoA* flanking homology regions into pBSANDdel
SG1348	pBSANDdel homology insertion check rev	ACGCATTGATTTGAGTCAGCT	Check for insertion of *oxdC-yvrJ-rsiO/sigO-rsoA* flanking homology regions into pBSANDdel
SG1349	sgRNA for *ΔoxdC-yvrJ-rsiO/sigO-rsoA* deletion fwd	TACGGTTCTCTGCAAGCCTTCATA	sgRNA to guide Cas9 in pBSANDdel for *ΔoxdC-yvrJ-rsiO/sigO-rsoA* deletion
SG1350	sgRNA for *ΔoxdC-yvrJ-rsiO/sigO-rsoA* deletion fwd	AAACTATGAAGGCTTGCAGAGAAC	sgRNA to guide Cas9 in pBSANDdel for *ΔoxdC-yvrJ-rsiO/sigO-rsoA* deletion
SG1382	MerRZntR^{A29E} + BsaI fwd	TTTAAA**GGTCTC**GTTTTgaggtGACAATATG GGGATGAAAA	Amplification of MerRZntR^{A29E} for Golden Gate assembly
SG1383	MerRZntR^{A29E} + BsaI rev	TTTAAA**GGTCTC**AGGGGTCAACAACCACT CTTAACGCCACT	Amplification of MerRZntR^{A29E} for Golden Gate assembly
SG1384	P_{merR} with 5’-Terminator + BsaI fwd	TTTAAA**GGTCTC**ACCCCAAAAACGCGAGC GCCTGACGCTCGCGTTTTTTAACGGAAGA ATGTGGCTTCTTGG	Amplification of P_{merR} to include 5’-Terminator
SG1385	P_{merR} with 5’-Terminator + BsaI rev	TTTAAA**GGTCTC**TATTCTGATTTTCATCCCC ATATTGTCACC	Amplification of P_{merR} to include 5’-Terminator
SG1387	P_{cadA} + BsaI Rev	TTTAAA**GGTCTC**ATACTGCGCTTGCTGTTT TTCATTGACACT	Amplification of P_{cadA}
SG1403	P_{cadA} + BsaI Fwd	TTTAAA**GGTCTC**ATACTATTCAGCTCCGTT TCCGTTGTTCT	Amplification of P_{cadA}
SG1388	P_{liaI} + BsaI fwd	TTTAAA**GGTCTC**ATACT*ATTGGCCAAAGCA GAAAGGTC*	Amplification of P_{liaI}
SG1389	P_{liaI} + BsaI rev	TTTAAA**GGTCTC**ATACT*ATTGGCCAAAGCA GAAAGGTC*	Amplification of P_{liaI}
SG1410	P_{xylA} + BsaI rev	TTTAAA**GGTCTC**CAAAATTCGATAAGCTTG GGATCCCAG	Amplification of P_{xylA}

[a] Restriction sites are in uppercase bold; QuickChange point mutation sites are underlined bold, RBS sequences in primers are in lowercase and underlined with a thin line; terminator sequence are underlined with a dotted line.

Supplementary Table S4. Relevant DNA sequences for plasmid construction

Part	Part type & Function	Sequence[a]
P_{merR20}	MerR Family promoter recognised by MerR and the chimeras derived from this study. Contains a 20-bp spacer between the -10 and -35 elements	AACGGAAGAATGTGGCTTCTTGGCGTGAAGAGCAGA<br>TATCTTTATTCTTAAACTTCTAAAAAAGCTTATGTGAA<br>CACTTGATAAATAAGGTTTTTTATCTGACCTATTTTAT<br>AAGATTATTCTATAAAAGAAAAAATAATGTATGAC**TT**<br>**GACC**GTGTACTATGGTACAGGGTT**TATACT**TTTTATT<br>GAGGTGACAATATGGGGATGAAAATCAGTGAATTGG<br>CTAAAGCGTGTGATGTGAATAAAGA
P_{merR19}	MerR Family promoter recognised by MerR and the chimeras derived from this study. Contains a 19-bp spacer between the -10 and -35 elements	AACGGAAGAATGTGGCTTCTTGGCGTGAAGAGCAGA<br>TATCTTTATTCTTAAACTTCTAAAAAAGCTTATGTGAA<br>CACTTGATAAATAAGGTTTTTTATCTGACCTATTTTAT<br>AAGATTATTCTATAAAAGAAAAAATAATGTATGAC**TT**<br>**GACC**GTGTACTATGGTACAGGGT**TATACT**TTTTATTG<br>AGGTGACAATATGGGGATGAAAATCAGTGAATTGGC<br>TAAAGCGTGTGATGTGAATAAAGA
P_{veg}	Strong constitutive promoter native to B. subtilis	GGAGTTCTGAGAATTGGTATGCCTTATAAGTCCAATT<br>AACAGTTGAAAACCTGCATAGGAGAGCTATGCGGGT<br>TTTTTATTTTACATAATGATACATAATTTACCGAAACTT<br>GCGGAACATAATTGAGGAATCATAGAATTTTGTCAAA<br>ATAATTTTATTGACAACGTCTTATTAACGTTGATATAA<br>TTTAAATTTTAT**TTGACA**AAAATGGGCTCGTGTTG**TA**<br>**CAAT**AAATGTAGT
MerR	Coding sequence for MerR including the native RBS and spacer region of MerR. Hg^{2}-sensor	gaggtgacaatATGGGGATGAAAATCAGTGAATTGGCT<br>AAAGCGTGTGATGTGAATAAAGAAACCGTTCGGTAT<br>TACGAGCGGAAAGGATTGATAGCCGGGCCTCCCAGA<br>AACGAATCAGGGTATCGAATATATTCAGAGGAAACA<br>GCAGATCGGGTACGGTTTATTAAACGAATGAAGGAA<br>TTGGATTTCTCGCTAAAGGAAATCCACCTGTTGTTTG<br>GTGTGGTTGATCAAGATGGGGAGAGATGTAAAGATA<br>TGTACGCCTTTACCGTTCAAAAAACCAAAGAAATCGA<br>GCGGAAAGTGCAGGGTTTGTTACGAATCCAACGGTT<br>ATTAGAGGAATTAAAAGAAAAGTGTCCAGATGAAAA<br>GGCGATGTATACCTGTCCTATTATTGAAACGTTAATG<br>GGAGGGCCTGATAAATAA
MerRZntR	Coding sequence for MerRZntR chimera including the native RBS and spacer region of MerR	gaggtgacaatATGGGGATGAAAATCAGTGAATTGGCT<br>AAAGCGTGTGATGTGAATAAAGAAACCGTTCGGTAT<br>TACGAGCGGAAAGGATTGATAGCCGGGCCTCCCAGA<br>AACGAATCAGGGTATCGACTATATACCGAAAGCGAT<br>CTCCAGCGATTGAAATTTATCCGCCATGCCAGACAAC<br>TAGGTTTCAGTCTGGAGTCGATCCGCGAGTTGCTGTC<br>GATCCGCATCGATCCTGAACACCATACCTGTCAGGAG<br>TCAAAAGGCATTGTGCAGGAAAGATTGCAGGAAGTC<br>GAAGCACGGATAGCCGAGTTGCAGAGTATGCAGCGT<br>TCCTTGCAACGCCTTAACGATGCCTGTTGTGGGACTG<br>CTCATAGCAGTGTTTATTGTTCGATTCTTGAAGCTCTT<br>GAACAAGGGGCGAGTGGCGTTAAGAGTGGTTGTTGA

MerRZntR^{A29E}	Coding sequence for MerRZntR chimera including the native RBS and spacer region of MerR. Contains a A29E substitution in the α-helix 2-3 loop region. Optimised Zn^{2+} - sensor	gaggtgacaatATGGGGATGAAAATCAGTGAATTGGCT<br>AAAGCGTGTGATGTGAATAAAGAAACCGTTCGGTAT<br>TACGAGCGGAAAGGATTGATAGAAGGGCCTCCCAGA<br>AACGAATCAGGGTATCGACTATATACCGAAAGCGAT<br>CTCCAGCGATTGAAATTTATCCGCCATGCCAGACAAC<br>TAGGTTTCAGTCTGGAGTCGATCCGCGAGTTGCTGTC<br>GATCCGCATCGATCCTGAACACCATACCTGTCAGGAG<br>TCAAAAGGCATTGTGCAGGAAAGATTGCAGGAAGTC<br>GAAGCACGGATAGCCGAGTTGCAGAGTATGCAGCGT<br>TCCTTGCAACGCCTTAACGATGCCTGTTGTGGGACTG<br>CTCATAGCAGTGTTTATTGTTCGATTCTTGAAGCTCTT<br>GAACAAGGGGCGAGTGGCGTTAAGAGTGGTTGTTGA
MerRZntR$^{A29E/G30H}$	Coding sequence for MerRZntR chimera including the native RBS and spacer region of MerR. Contains a A29E/G30H substitution in the α-helix 2-3 loop region	gaggtgacaatATGGGGATGAAAATCAGTGAATTGGCT<br>AAAGCGTGTGATGTGAATAAAGAAACCGTTCGGTAT<br>TACGAGCGGAAAGGATTGATAGAACATCCTCCCAGA<br>AACGAATCAGGGTATCGACTATATACCGAAAGCGAT<br>CTCCAGCGATTGAAATTTATCCGCCATGCCAGACAAC<br>TAGGTTTCAGTCTGGAGTCGATCCGCGAGTTGCTGTC<br>GATCCGCATCGATCCTGAACACCATACCTGTCAGGAG<br>TCAAAAGGCATTGTGCAGGAAAGATTGCAGGAAGTC<br>GAAGCACGGATAGCCGAGTTGCAGAGTATGCAGCGT<br>TCCTTGCAACGCCTTAACGATGCCTGTTGTGGGACTG<br>CTCATAGCAGTGTTTATTGTTCGATTCTTGAAGCTCTT<br>GAACAAGGGGCGAGTGGCGTTAAGAGTGGTTGTTGA
MerRZntR$^{A29E/G30H/P32V}$	Coding sequence for MerRZntR chimera including the native RBS and spacer region of MerR. Contains a A29E/G30H/P32V substitution in the α-helix 2-3 loop region	gaggtgacaatATGGGGATGAAAATCAGTGAATTGGCT<br>AAAGCGTGTGATGTGAATAAAGAAACCGTTCGGTAT<br>TACGAGCGGAAAGGATTGATAGAACATCCTGTTAGA<br>AACGAATCAGGGTATCGACTATATACCGAAAGCGAT<br>CTCCAGCGATTGAAATTTATCCGCCATGCCAGACAAC<br>TAGGTTTCAGTCTGGAGTCGATCCGCGAGTTGCTGTC<br>GATCCGCATCGATCCTGAACACCATACCTGTCAGGAG<br>TCAAAAGGCATTGTGCAGGAAAGATTGCAGGAAGTC<br>GAAGCACGGATAGCCGAGTTGCAGAGTATGCAGCGT<br>TCCTTGCAACGCCTTAACGATGCCTGTTGTGGGACTG<br>CTCATAGCAGTGTTTATTGTTCGATTCTTGAAGCTCTT<br>GAACAAGGGGCGAGTGGCGTTAAGAGTGGTTGTTGA
MerRCueR	Coding sequence for MerRCueR chimera including the native RBS and spacer region of MerR	gaggtgacaatATGGGGATGAAAATCAGTGAATTGGCT<br>AAAGCGTGTGATGTGAATAAAGAAACCGTTCGGTAT<br>TACGAGCGGAAAGGATTGATAGCCGGGCCTCCCAGA<br>AACGAATCAGGGTATCGCACCTACACGCAGCAGCAT<br>CTCAACGAACTGACCTTACTGCGCCAGGCACGGCAG<br>GTGGGCTTTAACCTGGAAGAGAGCGGCGAGCTGGTG<br>AATCTGTTTAACGACCCGCAGCGGCACAGCGCCGAC<br>GTCAAACGGCGCACGCTGGAGAAGGTGGCGGAGAT<br>CGAACGACACATTGAGGAGCTGCAATCCATGCGCGA<br>CCAGCTGCTGGCACTGGCGAATGCCTGCCCTGGCGA<br>TGACAGCGCCGACTGCCCGATTATCGAAAATCTCTCC<br>GGCTGCTGTCATCATCGGGCAGGGTGA

MerRCueR^{A29T}	Coding sequence for MerRCueR chimera including the native RBS and spacer region of MerR. Contains a A29T substitution in the α-helix 2-3 loop region	gaggtgacaatATGGGGATGAAAATCAGTGAATTGGCT<br>AAAGCGTGTGATGTGAATAAAGAAACCGTTCGGTAT<br>TACGAGCGGAAAGGATTGATAACAGGGCCTCCCAGA<br>AACGAATCAGGGTATCGCACCTACACGCAGCAGCAT<br>CTCAACGAACTGACCTTACTGCGCCAGGCACGGCAG<br>GTGGGCTTTAACCTGGAAGAGAGCGGCGAGCTGGTG<br>AATCTGTTTAACGACCCGCAGCGGCACAGCGCCGAC<br>GTCAAACGGCGCACGCTGGAGAAGGTGGCGGAGAT<br>CGAACGACACATTGAGGAGCTGCAATCCATGCGCGA<br>CCAGCTGCTGGCACTGGCGAATGCCTGCCCTGGCGA<br>TGACAGCGCCGACTGCCCGATTATCGAAAATCTCTCC<br>GGCTGCTGTCATCATCGGGCAGGGTGA
MerRCueR$^{A29T/G30P}$	Coding sequence for MerRCueR chimera including the native RBS and spacer region of MerR. Contains a A29T/G30P substitution in the α-helix 2-3 loop region	gaggtgacaatATGGGGATGAAAATCAGTGAATTGGCT<br>AAAGCGTGTGATGTGAATAAAGAAACCGTTCGGTAT<br>TACGAGCGGAAAGGATTGATAACACCGCCTCCCAGA<br>AACGAATCAGGGTATCGCACCTACACGCAGCAGCAT<br>CTCAACGAACTGACCTTACTGCGCCAGGCACGGCAG<br>GTGGGCTTTAACCTGGAAGAGAGCGGCGAGCTGGTG<br>AATCTGTTTAACGACCCGCAGCGGCACAGCGCCGAC<br>GTCAAACGGCGCACGCTGGAGAAGGTGGCGGAGAT<br>CGAACGACACATTGAGGAGCTGCAATCCATGCGCGA<br>CCAGCTGCTGGCACTGGCGAATGCCTGCCCTGGCGA<br>TGACAGCGCCGACTGCCCGATTATCGAAAATCTCTCC<br>GGCTGCTGTCATCATCGGGCAGGGTGA
MerRCueR$^{A29T/G30P/P32M}$	Coding sequence for MerRCueR chimera including the native RBS and spacer region of MerR. Contains a A29T/G30P/P32M substitution in the α-helix 2-3 loop region	gaggtgacaatATGGGGATGAAAATCAGTGAATTGGCT<br>AAAGCGTGTGATGTGAATAAAGAAACCGTTCGGTAT<br>TACGAGCGGAAAGGATTGATAACACCGCCTATGAGA<br>AACGAATCAGGGTATCGCACCTACACGCAGCAGCAT<br>CTCAACGAACTGACCTTACTGCGCCAGGCACGGCAG<br>GTGGGCTTTAACCTGGAAGAGAGCGGCGAGCTGGTG<br>AATCTGTTTAACGACCCGCAGCGGCACAGCGCCGAC<br>GTCAAACGGCGCACGCTGGAGAAGGTGGCGGAGAT<br>CGAACGACACATTGAGGAGCTGCAATCCATGCGCGA<br>CCAGCTGCTGGCACTGGCGAATGCCTGCCCTGGCGA<br>TGACAGCGCCGACTGCCCGATTATCGAAAATCTCTCC<br>GGCTGCTGTCATCATCGGGCAGGGTGA
RsoA	Coding sequence for RsoA, co-σ-factor of SigO	GTGGACGGCCAGTTTGAACAAAAAAAGAAACAAAAA<br>GACGAGACTTATGACATTGAGCACCTGATTGCATGCT<br>TTTCACCGATGATCAGA<br>AAAAAACTCAGCAATACGTCCTATCAAGAAAGAGAA<br>GATTTAGAGCAAGAGCTGAAGATCAAAATGTTTGAA<br>AAGGCTGATATGCTTTTATGTCAGGATGTACCGGGGT<br>TTTGGGAGTTTATTTTGTACATGGTAGATGAGAACTC<br>ATAA
SigO	Coding sequence for SigO, co-σ-factor of RsoA	ATGAAGCATCCCATCGTGAAGCATTTTTTGAGCAATC<br>CTCAGCATTACCGTTTGTTCAAAAACGTAATGGAAAG<br>CCCTAACGAAAAAGATGCAAGATCATTGGACGAGCT<br>ATTTAAGCAATTTTATAAAGAAATCCGCATCGTCAAG

		TATATGAATTCAATGATTCGCATCTTTTCTATTGATTTT<br>GATAAGCGGGTTCGCAAAAACCAAAAACGGTATCCA<br>CTGACGGTTGATCATCCGGAGGCGGGAGATCGGCTT<br>TCTTCCGAAACAGGTAGCGATGCATTTGAAGAATTTT<br>TAGACAGGCAGGATGATTTGAGCCAGCATGTACAGG<br>ATTACCAGCTCTACCAAGCGATCCAGAAGCTGACTGA<br>CAAACAAAAAAGTGTGCTGACGAAAGTCTATCTTCAC<br>GGTGCCACGATGCAGGAGATTGCAGATTCATTAGGG<br>GAGTCCCGACAAAACATCTCCAACATTCATAAAAAGG<br>GGCTGGAGAATATCAGAAAGCAGTTAGCGGCGCAAA<br>AAAAGGGGGAAAAGTAA
P_{oxdC}	P_{oxdC} promoter recognised by SigO and RsoA	GATTCGAAAAGAAGTTTGATCAACTAATAGAACTAAT<br>GACAGAACTGAAAGATCATGCAAAAAAATAATTTTTC<br>AATCGAAG**TTGACT**TTTCACTGGTTTTTT**TCACTTA**AC<br>AAAACAGAAGGGAAAACGAA
P_{xylA}	Xylose-inducible promoter lacking CRE-element. Sequence is derived from Radeck *et al* [3]	AAGGCCAAAAAACTGCTGCCTTCGGATCAGCGATATC<br>CACTTCATCCACTCCATTTGTTTAATCTTTAAATTAAGT<br>ATCAACATAGTACATAGCGAATCTTCCCTTTATTATAT<br>CTAATGTGTTCATAAAAAACTAAAAAAAA**TATTGA**AA<br>ATACTGACGAGGTTA**TATAAG**ATGAAAATAAGTTAG<br>TTTGTTTAAACAACAAACTAATAGGTGATGTACTTACT<br>ATATGAAATAAAATGCATCTGGGATCCCAAGCTTATC<br>GAA
P_{liaI}	Bacitracin-inducible promoter. Sequence is derived from Radeck *et al* [3]	ATTGGCCAAAGCAGAAAGGTCCGACCTAATTAAAGA<br>AAGGGAAGCAAGTGTTCATCTGTAAAGGGTTTTAAA<br>ACGCCATGCCTCGTGCATGGCGTTTTTTTGTGCCAAT<br>GGGTCCGGTGCGAGATACGACTCCGGTCTTATATAAA<br>AATCAATCTCTGAT**TCG**TTTTGCATATCTTCCAACTTG<br>**TATAAG**ATGAAGACAAGGAAAACGA
Terminator 1 "Term 1"	Strong minimal terminator to prevent transcriptional read-through. Sequence is derived from Cui *et al* [5]	AAAAACGCGAGCGCCTGACGCTCGCGTTTTTT
RBS R1	RBS used for SigO and RsoA translation. Sequence is derived from Guiziou *et al* [6]	GCTCTTAAAGGGGGTTTTAGA
P_{cadA}	Metal inducible promoter regulated natively by CzrA	ATTCAGCTCCGTTTCCGTTGTTCTGAATGCTCTT<br>CGTCTGCAAAAAGTAAAATGAAAAACCGGCTATA<br>TGCCGGTTTTTGTTTTTCA**TTGACA**CTTTCTTGG<br>AAAACAACA**TATAAT**AGGTGTAACTTATATATGA<br>GTATATGCTCATATATATAAAATAAATACAATAC<br>TCATTGATACGCTTTGAAGAGGGAA

[a] For sequences of this study, the -10 and -35 elements are in bold; positions where a regulator binds are underlined; RBS and spacer are in lowercase.

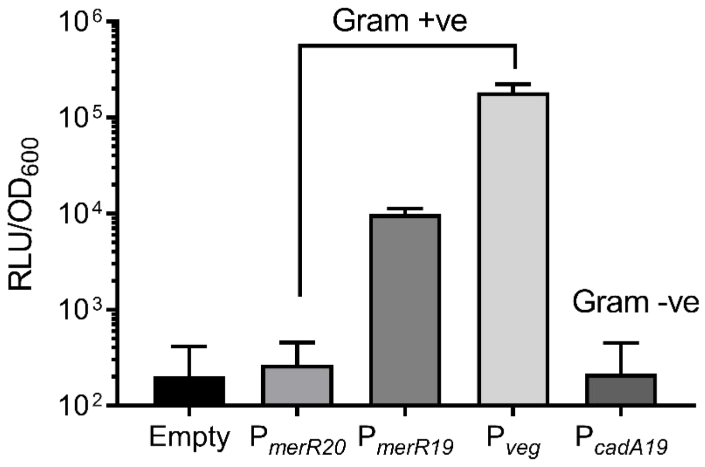

Supplementary Figure S1. Comparison of Gram +ve and Gram -ve MerR promoter activity in *B. subtilis*. Cells harbouring either an empty luciferase reporter, P_{merR20} (Gram +ve), P_{merR19} (Gram +ve), P_{veg} (Gram +ve) or P_{cadA19} (Gram-ve) were grown in MM9 media with luciferase activity measured overtime. Values presented are the average of three time points (35-, 40- and 45-minutes) following initial inoculation into MM9 medium. The strong Gram +ve *B. subtilis* promoter P_{veg}, was included as a control. Subscripts indicate the size of the spacer region between the -10 and -35 elements for MerR family promoters. Data are the ± standard deviation of triplicate measurements performed on three different days.

A)

```
ZntR_Ec   MYRIGELAKMAEVTPDTIRYYEKQQMMEHEVRTEGGFRLYTESDLQRLKFIRHARQLGFS 60
ZntR_Se   MYRIGELAKMAEVTPDTIRYYEKQQMMEHEVRTEGGFRLYTESDLQRLKFIRHARQLGFS 60
ZntR_Kp   MYRIGELAKMAEVTPDTIRYYEKQQMMEHEVRTEGGFRLYTESDLQRLKFIRHARQLGFS 60
ZntR_Sf   MYRIGELAKMAEVTPDTIRYYEKQQMMEHEVRTEGGFRLYTESDLQRLKFIRHARQLGFS 60
ZntR_Pa   MYRIGELAKLANVTPDTIRYYEKQQMIDHEVRTEGGFRLYTDNDLQRLRFIRYARQLGFT 60
          *********:*:**************::*************:.*****:***:******:
```

B)

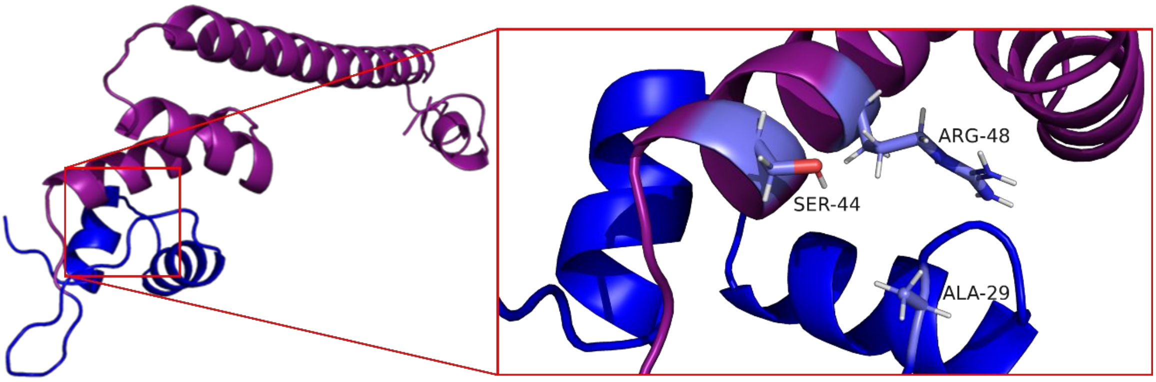

Supplementary Figure S2. Comparison of ZntR sequences and structural analysis of the chimera MerRZntR. A) Sequence alignment of ZntR homologues from various Gram-negative genetic backgrounds ("Ec" – *E. coli*, accession code: AAC76317.1; "Se"- *S. enterica*, accession code: EBW6030787.1; "Kp" – *K. pneumoniae*, accession code: CDK70471.1; "Sf" – *S. flexerni*, accession code: EAA3112577.1; "Pa" – *P. aeruginosa*, accession code: MBH4409345.1). Residues of interest involved in interdomain communication are highlighted in bold purple. Asterisk " * " indicates fully conserved residues, colon " : " indicates conserved residues with similar properties, and period " . " indicates residues of weakly similar properties. **B)** Structural analysis of the residues between α-helices 2-3 in MerRZntR. Here, the MerR (*S. aureus*) derived DNA-Binding Domain is coloured dark blue, whilst the ZntR (*E. coli*) derived Metal-Binding Domain is coloured purple. Residues of interest are coloured lavender blue and are numbered accordingly. Due to the presence of the non-polar residue Ala-29, no hydrogen bonding is present between Ala-29 (DNA-Binding Domain) and Ser-44 and Arg-48 (Metal Binding Domain).

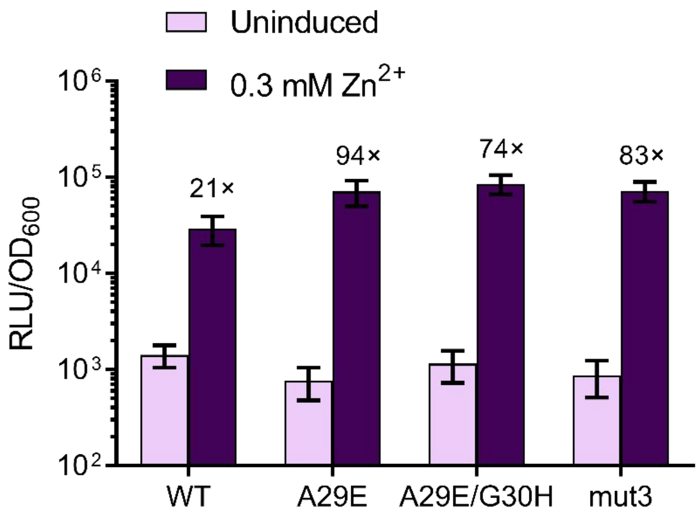

Supplementary Figure S3. Activity of double and triple MerRZntR mutants against the wild-type and single mutant MerRZntR^{A29E}. Residues in between alpha-helix 2-3 were mutated to those found natively in ZntR, generating MerRZntR$^{A29E/G30H}$ and MerRZntR$^{A29E/G30H/P32V}$ (mut3), with the activity compared relative to both wild-type (MerRZntR) and the single mutant (MerRZntR^{A29E}). Cells were grown to OD_{600}= ~ 0.03 and induced at the highest sub-lethal tested concentration of Zn^{2+} with luciferase activity (relative luminescence units (RLU) normalised by cell density (OD_{600})) for three time points (35-, 40- and 45-mins) post induction. Fold-induction values of the induced (dark purple) are relative to the respective uninduced strain (light purple). Values are presented as mean and ± standard deviation of either two or three independent replicates.

A)

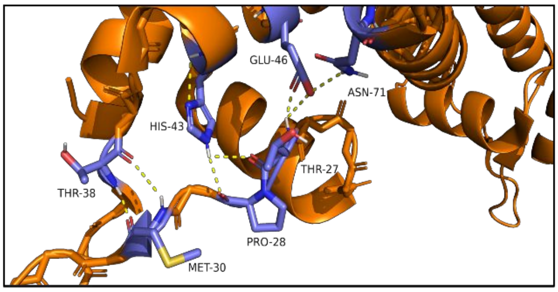

B)

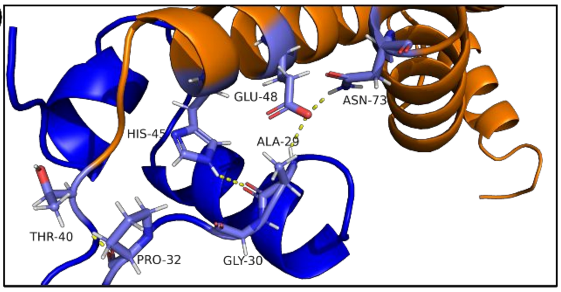

C)

```
CueR_Ec   MNISDVAKITGLTSKAIRFYEEKGLVTPPMRSENGYRTYTQQHLNELTLLRQARQVGFNL 60
CueR_Kp   MNISDVAKITGLTSKAIRFYEEKGLVTPPMRSENGYRTYTQQHLNELTLLRQARQVGFNL 60
CueR_Sf   MNISDVAKITGLTSKAIRFYEEKGLVTPPMRSENGYRTYTQQHLNELTLLRQARQVGFNL 60
CueR_Pa   MNISDVAKKTGLTSKAIRFYEEKGLVTPPLRSENGYRTYSQQHLDELTLLRQARQVGFNL 60
CueR_Se   MNISDVAKKTGLTSKAIRFYEEKGLVTPPLRSENGYRTYTQKHLNELTLLRQARQVGFNL 60
          ******** ********************:*********:*:**:***************
```

Supplementary Figure S4. Comparison of inter-domain communication between CueR and the chimera MerRCueR.A) Magnification of the CueR (*E. coli*, accession code: CAD6020341.1) inter-domain hydrogen bonding network between α-helices 2-3. **B)** Magnification of the MerRCueR chimera inter-domain hydrogen bonding network between α-helices 2-3. Here, the MerR (*S. aureus*) derived DNA-Binding Domain is indicated in dark blue, whilst the CueR (*E. coli*) derived Metal-Binding Domain is indicated in orange. For panels **A** and **B,** residues of interest are coloured in lavender blue and are numbered accordingly, with hydrogen bonds indicated in yellow. **C)** Sequence alignment of CueR from various Gram-negative genetic backgrounds ("Ec" – *E. coli*, accession code: CAD6020341.1; ; "Kp" – K. pneumoniae, accession code: OZQ58601.1; "Sf" – *S. flexerni*, accession code: EFX2973845.1; "Pa" – *P. aeruginosa*, accession code: MXH36715.1; "Se"- *S. enterica*, accession code: EAS1883030.1). Residues of interest involved in interdomain communication are highlighted in bold orange. Asterisk " * " indicates fully conserved residues, colon " : " indicates conserved residues with similar properties, and period " . " indicates residues of weakly similar properties.

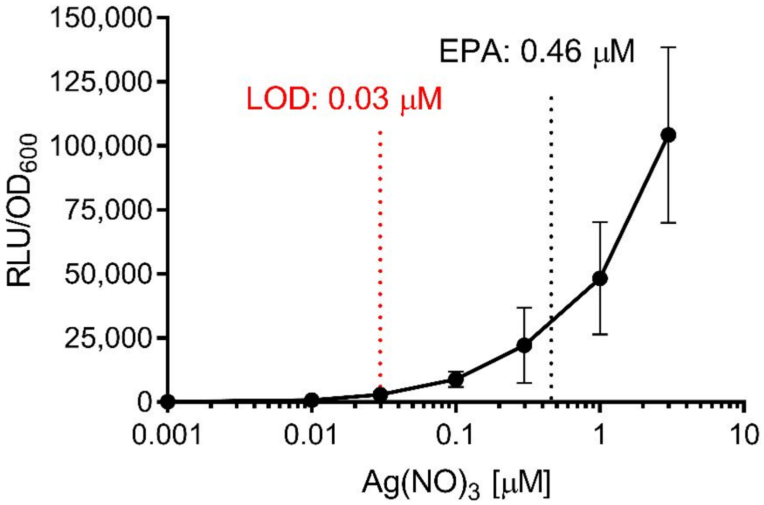

Supplementary Figure S5. Dose response of P_{merR} regulated by MerRCueR^{mut3} in response to Ag^{+} induction. Transcriptional output from P_{merR} is shown in response to various concentrations of Ag^{+}. Cells were induced at OD_{600} = ~ 0.03 with luciferase activity (relative luminescence units [RLU]) normalised to optical density (OD_{600}) values (RLU/OD_{600}) from three time points (35-, 40- and 45-mins post induction). Values for the limit of detection (LOD) and Environmental Protection Agency (EPA) guideline values are indicated. Values are presented as mean and ± standard deviation of either two or three independent replicates.

-35 -10

P_{merR20} ATGTATGAC**TTGACC**GTGTACTATGGTACAGGGTT**TATACT**TTTTATT

P_{merR19} ATGTATGAC**TTGACC**GTGTACTATGGTACAGGGT**TATACT**TTTTATT

Supplementary Figure S6. Wild-type (P_{merR20}) and mutant (P_{merR19}) promoters. The wild-type P_{merR20} promoter was used as a template for targeted mutagenesis to remove 1 bp adjacent to the -10 element (indicated by red arrow), as done previously by Parkhill *et al* [7] to generate the mutant promoter P_{merR19}. The MerR dyad sequence is underlined with both the -35 and -10 element in bold.

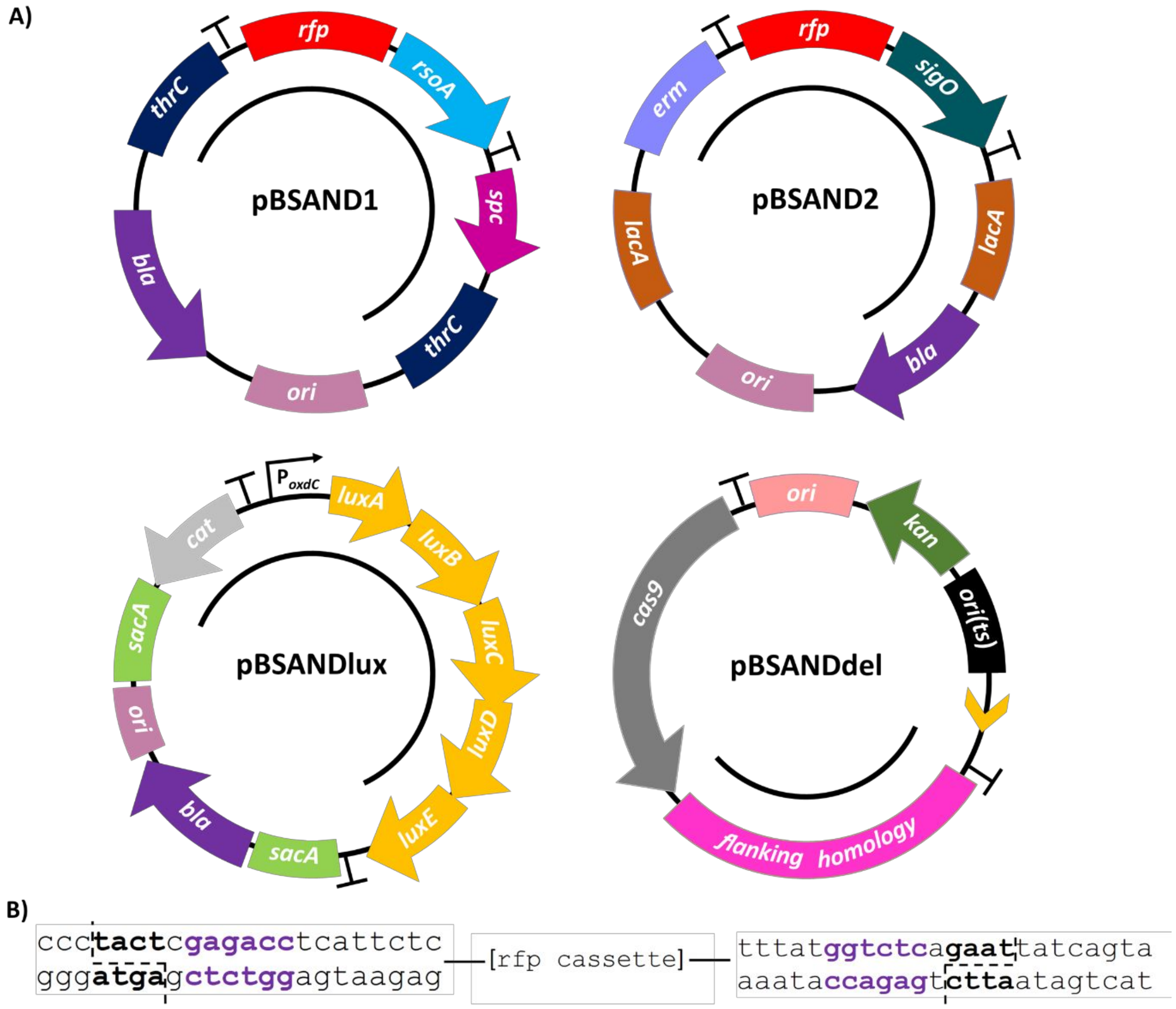

Supplementary Figure S7. Maps of the *B. subtilis* SANDBOX plasmids. A) Vector architecture for plasmids pBSAND1 and pBSAND2 both of which contain one half of the two-subunit sigma factor system SigO-RsoA. Plasmids pBSAND1, pBSAND2 and pBSANDlux are all integrative vectors with resistance markers *spc* (spectinomycin), *erm* (MLS; *macrolide, lincosamide and streptogramin B antibiotics if induced by erythromycin)* and cat (chloramphenicol) and integrate at the loci *thrC, lacA* and *sacA*, respectively. Whilst pBSANDdel is an integrative vector, the flanking homology region (shown in pink) is the only integrative portion of the plasmid. The gRNA to cut within the *sigO-rsoA* regulon is indicated with an orange arrowhead. Plasmid pBSANDlux is a luciferase-based reporter vector (P_{oxdC}-*luxABCDE*) and pBSANDdel is a modified CRISPR-Cas9 vector designed to knockout the SigO-RsoA regulon. The integrative portion of all the logic gate plasmids are shown with a black line, terminators are indicated with the "T" symbol, and all comprise a *bla* (ampicillin) resistance marker to allow for selection in *E. coli* – the exception of which is pBSANDdel which has a *kan* (kanamycin) marker for selection in both *E. coli* and *B. subtilis*. Plasmids pBSAND1, pBSAND2, pBSANDlux and pBSANDdel are derived from pBS4S, pBS2E, pBS3Clux and pJOE8999 [3,4] . **B)** The Golden Gate cloning site based on BsaI. The RFP cassette is flanked by two Golden Gate restriction sites, highlighted in bold purple, with the overhang indicated in bold black.

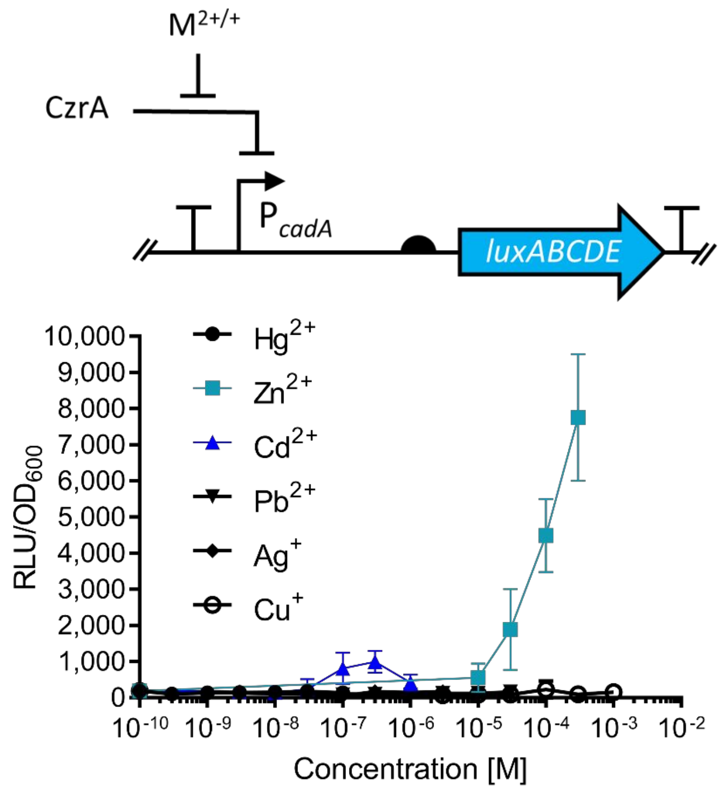

Supplementary Figure S8. *Bacillus subtilis* metal-sensory circuit controlled by the native CzrA regulator. In the circuit shown, CzrA mediated repression of the cognate promoter P_{cadA} is relieved upon the addition of heavy metal ions. Transcriptional output from P_{cadA}, measured via luciferase activity (luxABCDE, light blue arrow) is shown in response to various concentrations of heavy metals. Inducers, Zn^{2+} and Cd^{2+} are indicated in teal and dark blue respectively. $M^{+/2+}$ indicates the addition of either a monovalent or divalent metal ion. Cells were induced at OD_{600} = ~ 0.03 with luciferase activity (relative luminescence units [RLU]) normalised to optical density (OD_{600}) values (RLU/OD_{600}) from three time points (35-, 40- and 45-mins post induction). Values are presented as mean and ± standard deviation of either two or three independent replicates.

References

1. Derre, I., Rapoport, G. & Msadek, T. The CtsR regulator of stress response is active as a dimer and specifically degraded in vivo at 37°c. *Mol. Microbiol.* **38**, 335–347 (2000).
2. Schmalisch, M. *et al.* Small genes under sporulation control in the Bacillus subtilis genome. *J. Bacteriol.* **192**, 5402–5412 (2010).
3. Radeck, J. *et al.* The Bacillus BioBrick Box: Generation and evaluation of essential genetic building blocks for standardized work with Bacillus subtilis. *J. Biol. Eng.* **7**, (2013).
4. Altenbuchner, J. Editing of the Bacillus subtilis genome by the CRISPR-Cas9 system. *Appl. Environ. Microbiol.* **82**, 5421–5427 (2016).
5. Cui, W. *et al.* Data-Driven and in Silico-Assisted Design of Broad Host-Range Minimal Intrinsic Terminators Adapted for Bacteria. *ACS Synth. Biol.* **10**, 1438–1450 (2021).
6. Guiziou, S. *et al.* A part toolbox to tune genetic expression in Bacillus subtilis. *Nucleic Acids Res.* **44**, 7495–7508 (2016).
7. Parkhill, J. & Brown, N. L. Site-specific insertion and deletion mutants in the mer promoter-operator region of Tn501; the nineteen base-pair spacer is essential for normal induction of the promoter by MerR. **18**, 5157–5162 (1990).
